# A Nasal Vaccine Displaying Anthrax Antigen on the Surface of *Lactiplantibacillus plantarum* Induces Protective Mucosal Immunity against Anthrax Toxin

**DOI:** 10.4014/jmb.2503.03036

**Published:** 2025-05-26

**Authors:** In-Hwan Jang, In Ryeong Jung, Hyojung Suh, Hyo-Joo Ahn, Donghyun Kim, Youn Soo Choi, June-Yong Lee, Kyung-Ah Lee

**Affiliations:** 1 Department of Microbiology & Immunology, Seoul National University College of Medicine, Seoul 03080, Republic of Korea; 2 Department of Microbiology and Immunology, Yonsei University College of Medicine, Seoul 03722, Republic of Korea; 3 Brain Korea 21 PLUS Project for Medical Sciences, Yonsei University College of Medicine, Seoul 03722, Republic of Korea; 4 Department of Biomedical Sciences, Seoul National University College of Medicine, Seoul, 03080 Republic of Korea; 5 Saeloun Bio Inc., Seoul 08826, Republic of Korea; 6 Institute of Endemic Diseases, Seoul National University Medical Research Center, Seoul 03080, Republic of Korea; 7 Department of Medicine, Seoul National University College of Medicine, Seoul 08826, Republic of Korea; 8 Transplantation Research Institute, Seoul National University Hospital, Seoul 08826, Republic of Korea; 9 Institute for Immunology and Immunological Diseases, Yonsei University College of Medicine, Seoul 03722, Republic of Korea

**Keywords:** Anthrax, nasal vaccine, *Lactiplantibacillus plantarum*, protective antigen

## Abstract

Anthrax, particularly inhalational anthrax, poses a severe threat due to its high fatality rate and potential misuse as a biological weapon. Current anthrax vaccines require multiple injections, which limits their practicality and underscores the need for improved, user-friendly vaccination strategies. In this study, we developed a novel nasal booster vaccine platform aimed at preventing inhalational anthrax by inducing mucosal immunity at the pathogen's entry site, the respiratory tract. We successfully engineered a biologically safe *Lactiplantibacillus plantarum* (*L. plantarum*) strain to display the anthrax protective antigen (PA) on its surface. Following systemic priming by intramuscular immunization, nasal boosting with this recombinant *L. plantarum* strain significantly enhanced protective antigen (PA)-specific mucosal IgA production in both nasal and bronchoalveolar lavage fluids. Importantly, mucosal IgA antibodies elicited through this nasal boosting strategy effectively neutralized anthrax lethal toxin. Our findings indicate that recombinant *L. plantarum* expressing PA represents a promising nasal vaccine booster platform, potentially replacing traditional intramuscular boosters, and paving the way for the development of effective and easily administered mucosal vaccines against anthrax and other respiratory pathogens.

## Introduction

Anthrax, caused by *Bacillus anthracis*, is a significant threat due to its high mortality rate and potential use as a biological weapon [1]. Its spores are resilient, easily disseminated, and notably were weaponized during the 2001 bioterrorism attacks in the United States, highlighting the urgent need for effective preventive strategies such as vaccination [2]. Inhalational anthrax, the most severe form of anthrax infection caused by *B. anthracis*, has a mortality rate exceeding 90% without prompt treatment [2]. Due to rapid disease progression and the limited efficacy of antibiotics once severe symptoms emerge, vaccination remains the most effective preventive measure [3]. Moreover, anthrax spores can persist in environmental conditions for decades, posing prolonged risks of exposure [4]. High-risk groups, including military personnel, laboratory researchers, veterinarians, and emergency responders, necessitate vaccination to mitigate such risks. Furthermore, an outbreak could result in devastating economic and public health consequences, underscoring the critical need for efficient vaccine strategies. Current anthrax vaccines, such as the FDA-approved Anthrax Vaccine Adsorbed (AVA), the UK's Anthrax Vaccine Precipitated (AVP), and live attenuated vaccines utilized in countries like Russia and China, require multiple injections and regular booster doses, significantly limiting their practicality during public health emergencies [5-9]. Thus, the development of improved vaccination strategies is imperative for global health security.

Mucosal vaccines, particularly those administered via the nasal route, represent a promising alternative to traditional intramuscular vaccines due to their capacity to induce robust mucosal immunity in addition to systemic responses [10, 11]. Secretory IgA antibodies generated by nasal immunization effectively neutralize pathogens at entry sites, providing frontline protection crucial against respiratory pathogens such as anthrax [10-13]. Furthermore, recent studies highlight the efficacy of nasal vaccination in inducing broad and durable protection against respiratory pathogens, demonstrated by successful mucosal vaccines against respiratory pathogens such as SARS-CoV-2 and related sarbecoviruses [12, 13]. Moreover, nasal vaccination facilitates easier administration, potentially enhancing compliance and accessibility during large-scale vaccination campaigns or pandemics.

Among various delivery platforms, *Lactiplantibacillus plantarum* has emerged as a highly promising vector. *L. plantarum* is an FDA-recognized probiotic organism with well-established safety profiles, offering distinct advantages in terms of stability, storage, and transport without stringent cold-chain requirements [14, 15]. Recent studies have highlighted the capability of recombinant *L. plantarum* strains to effectively express and deliver antigens at mucosal surfaces, enhancing immunogenicity and promoting both systemic and mucosal antibody responses [14, 15]. Notably, the cell-wall anchoring mechanism of *L. plantarum* stabilizes antigen expression, minimizing antigen degradation at mucosal surfaces and thereby effectively enhancing vaccine efficacy following nasal administration [15].

In this study, we aimed to develop an efficient anthrax vaccine delivery vector utilizing *L. plantarum* to express anthrax antigens, assessing its capacity to induce protective mucosal and systemic immunity through nasal immunization. This approach seeks to overcome current limitations of existing anthrax vaccines and provide enhanced protection against inhalational anthrax and other respiratory pathogens.

## Materials and Methods

### Bacterial Strains and Growth Conditions

*E. coli* was cultured at 37°C with agitation in Luria Bertani (LB) broth. *L. plantarum* WJL was grown in MRS broth at 37°C without agitation. When necessary, culture media were supplemented with antibiotics at the following concentrations: kanamycin 50 μg/ml for *E. coli*, erythromycin 200 μg/ml for *E. coli* and erythromycin 10 μg/ml for *L. plantarum* WJL.

### Plasmid Construction and DNA Manipulation

**Fig. 1:**
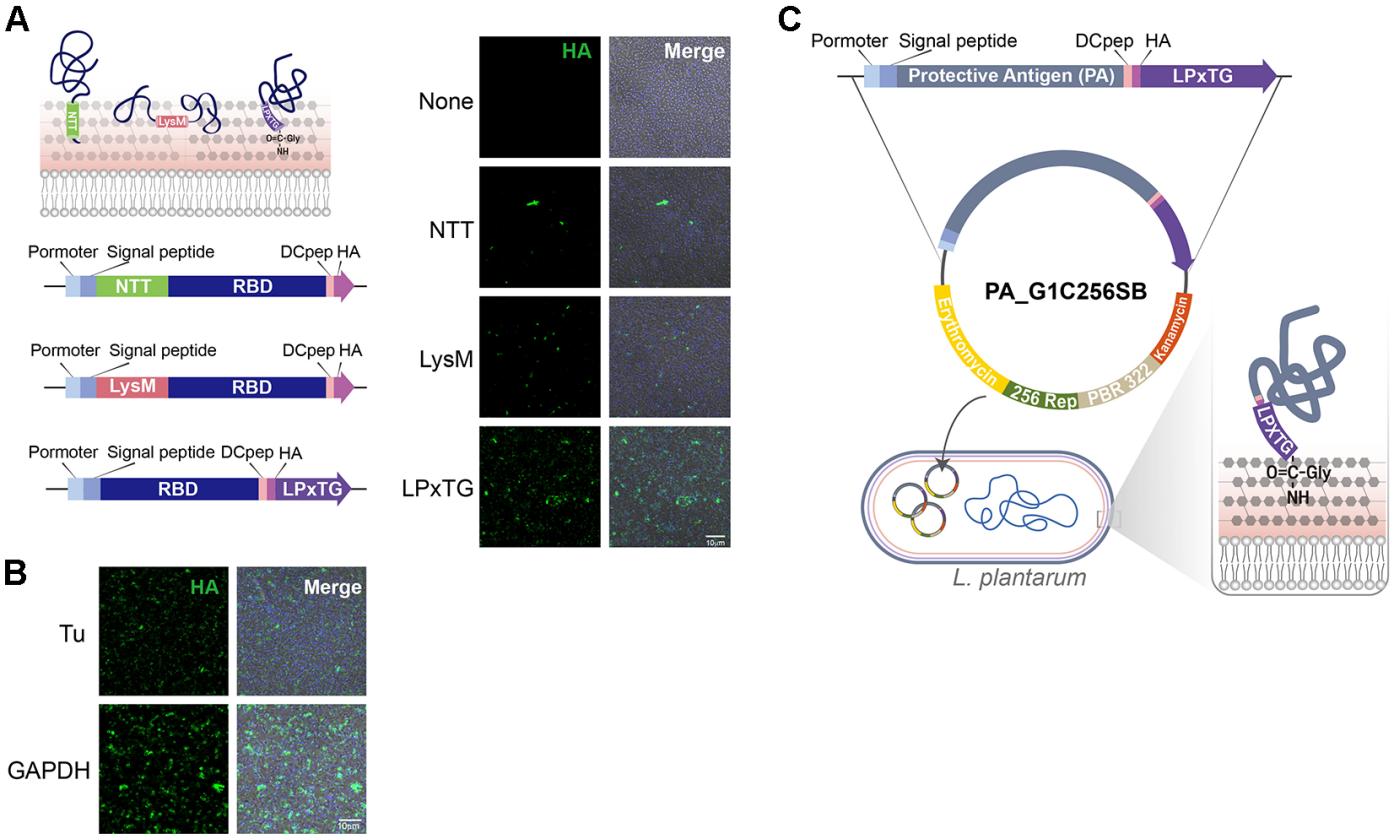
Optimization of anchor systems and promoters for antigen display on the surface of *Lactiplantibacillus plantarum*. (**A**) Schematic representation and comparison of bacterial surface anchor systems (N-terminal transmembrane [NTT], LysM, and LPxTG) for displaying the receptor-binding domain (RBD) of SARS-CoV-2 fused to an HA tag (HA-RBD). Immunofluorescence analysis demonstrated that the LPxTG anchor system provided superior stability and surface expression efficiency of HA-RBD compared to the NTT and LysM anchors. HA-tagged proteins were detected using anti-HA antibodies (green), and bacterial nuclei were visualized with DAPI (blue). Scale bar: 10 μm. (**B**) Comparative evaluation of promoter efficiency for antigen expression in *L. plantarum*. The laboratory-optimized GAPDH promoter exhibited significantly higher levels of HA-RBD expression compared to the elongation factor Tu promoter, as shown by immunofluorescence staining. Anti- HA staining (green) highlights antigen expression, while nuclei are stained with DAPI (blue). Scale bar: 10 μm. (**C**) Schematic illustration of the final recombinant vector construction for expressing anthrax protective antigen (PA) in *L. plantarum*. The full-length PA gene was codon-optimized to enhance expression efficiency in *L. plantarum* and fused at its C-terminal end with a dendritic cell-targeting peptide (DCpep), an HA epitope tag, and the LPxTG anchor motif.

To generate the vector 256SB (Fig. 1C), the PBR322 origin and a kanamycin resistance gene were amplified from pET28a (#69864 Novagen, Germany) and pENTR D-TOPO (Thermo Fisher Scientific, #K240020SP, USA), respectively, and assembled by PCR. The assembled PCR product was then double-digested with NotI and SphI, and ligated with a 600 bp fragment of the LacZ region containing the multi-cloning site from pBluescript SK (Agilent, USA). The p256 origin was amplified from pCD256AEc-Ptuf34-mCherry using SphI and NheI, and subsequently inserted into the intermediate vector containing the PBR322 origin and kanamycin resistance gene [16]. Finally, the erythromycin resistance gene, amplified from pSip411, was inserted between the NheI and SalI sites of the vector containing the p256 origin, PBR322 origin, and kanamycin resistance gene[17].To evaluate the efficiency of antigen display, three anchor systems were tested: the N-terminal transmembrane (NTT) anchor [18], the LysM anchor (both N-terminal) [19], and the LPxTG anchor (C-terminal) [20]. Constructs expressing the SARS-CoV-2 receptor-binding domain (RBD, GenBank: MN908947) fused to an HA tag were designed using each anchor type (Fig. 1A). Expression of fusion proteins in *L. plantarum* was driven by either the elongation factor Tu promoter or a laboratory-optimized GAPDH promoter. To eliminate a plasmid instability element within the GAPDH promoter, the promoter was serially truncated in 10 bp increments, from its full length of 200 bp down to 70 bp, using PCR. The optimal version of the GAPDH promoter was determined to be the 70 bp 5’ UTR of *L. plantarum* WJL_1077. Our experiments to develop genetically modified organisms were approved (23-RDM-010) from Korea disease control and prevention agency.

To display the PA protein on the surface of *L. plantarum*, the PA sequence (ACCESSION No. P13423, 2205 bp) was used to design a synthetic gene codon-optimized for *L. plantarum* expression. PCR fragments amplified from the synthetic PA gene were cloned between the signal peptide and the DC pep-HA-LPxTG cassette of the G1C256SB vector, which includes: Signal peptide (MRRKLVGYMLSMLTVILALFMLGSTAHAKE) for protein secretion, DC pep (FYPSYHST- PQRP) to enhance immunogenicity by targeting dendritic cells, HA tag (YPYDVPDYA), LPXTG (*L. plantarum* WCSF1 lp_25) (Fig. 1C). The PA-LPxTG cassette was expressed under the control of the GAPDH promoter.

### Recombinant Proteins

To produce recombinant anthrax protective antigen (PA) protein, a 2205-nucleotide sequence encoding amino acids 30–764 of *B. anthracis* PA was obtained by PCR amplification using PA_G1C256SB as a template. The primers for the amplification were PA_BamHI_Fw: CGGGATCCGAAGTTAAGCAAGAAAACCGTTTA and PA_XhoI_Rv: CCGCTCGAGACCGATTTCGTAACCCTTCTTTGAG. His-tagged PA was generated by cloning the PCR product into the BamHI and XhoI sites of the vector pET28a (Novagen, #69864) and transforming it into *E. coli* BL21. To express the recombinant PA protein, a 1 L culture of bacterial cells harboring PA_pET28a was grown until the optical density reached OD^600^ 0.6 and then induced with 1 mM isopropyl-β-D-thiogalactopyranoside at 25°C for 16 h. The cultured cells were centrifuged and lysed using HisTALON xTractor buffer (Clontech, #635651, USA). His-tagged PA was purified using TALON metal affinity resin (Clontech, #635502), followed by dialysis with 1× TBS buffer containing 5% glycerol. The quality of the recombinant PA protein was confirmed by SDS-PAGE analysis.

Recombinant lethal factor (LF) protein was kindly provided by Dr. Dong Hyun Song (Agency for Defense Development, ADD).

### Protein Extraction and Western Blot

To isolate the cell wall fraction from *L. plantarum*, 10 ml of bacteria were cultured in MRS medium at 37°C until reaching the stationary phase. The bacterial cells were collected by centrifugation, washed once with PBS, and disrupted using bead beating (6,500 rpm, 3 × 40 sec) with 0.1 mm silica beads to generate cell wall fraction. The disrupted cells were centrifuged at 1,000 ×*g* for 1 min, and the resulting supernatant was further centrifuged at 16,000 ×*g* for 30 min at 4°C to pellet the cell wall fraction. For whole-cell lysate preparation, 1 mL of bacterial culture at 37°C (stationary phase) was centrifuged to collect the cell pellet. Both the whole-cell pellet and cell wall fraction were resuspended in SDS-PAGE sample buffer. The samples were boiled for 7 min, and the supernatant was recovered. The expression of recombinant PA protein was analyzed by SDS-PAGE, followed by Western blotting. The following primary antibodies were used to assess protein expression levels: Anti-HA tag antibody (1:1000, Abcam, ab9110, UK), Anti-protective antigen antibody (1:1000, Abcam, ab69485).

### Immunofluorescence Staining

*L. plantarum* was cultured overnight in MRS medium. Bacterial cells (10^8^ cells/tube) were harvested by centrifugation at 5,000 ×*g* for 5 min and washed twice with PBS. For fixation, the cell pellet was incubated in 4%paraformaldehyde at room temperature for 10 min, followed by two additional PBS washes to remove excess fixative. The cells were then resuspended in 5% bovine serum albumin (BSA) in PBS and incubated for 1 h to block nonspecific binding. The samples were washed twice with PBS and incubated overnight at 4°C with the primary antibody diluted in 3% BSA in PBS. Following primary antibody incubation, the cells were washed three times for 5 min each with 0.1% Triton X-100 in PBS, then incubated with the secondary antibody and DAPI in 3% BSA in PBS for 20 min at room temperature. The samples were washed three more times for 5 min each with 0.1% Triton X-100 in PBS, followed by two additional washes with 1× PBS for 5 min each. Finally, the samples were resuspended in 300–400 μl of PBS, mounted onto a confocal dish (SPL; 100350) and analyzed using confocal microscopy (Carl Zeiss; LSM700, Germany). The following antibodies and reagents were used in this study: Primary antibodies: Anti-HA (1:500; Abcam, ab9110) and Anti-PA (1:500; Abcam, ab69485). Secondary antibodies: Alexa Fluor 568 goat anti-mouse IgG (1:500; Invitrogen, A11004, USA) and Alexa Fluor 488 goat anti-rabbit IgG (1:500; Invitrogen, A11008). Fluorescent dye: DAPI (1:1000; Roche, 10236276001, Switzerland) was used for nuclear staining.

### Mouse Immunization and Sample Collection

Seven-week-old female C57BL/6J mice were immunized intramuscularly with 5 μg purified recombinant PA protein adsorbed to AddaVax (InvivoGen). Two weeks after priming, mice received intranasal boosting for two consecutive days with either 1 × 10^9^ CFU of recombinant LP-PA or control LP. Serum, bronchoalveolar lavage fluid (BALF) and mediastinal lymph nodes were harvested two weeks after the final immunization. Blood was collected from the retro-orbital sinus under anesthesia and centrifuged at 10,000 rpm for 60 min to obtain serum. BALF samples were directly collected for mucosal antibody analysis. All experiments were performed according to the guidelines approved by the Institutional Animal Care and Use Committee (IACUC) of Seoul National University (SNU-231226-1-4) and Yonsei University College of Medicine (2023-0135).

### ELISA Analysis

Enzyme-linked immunosorbent assays (ELISA) were conducted to quantify antigen-specific antibody responses. Briefly, 96-well ELISA plates (Greiner Bio-One, 655061, Austria) were coated overnight at 4°C with either 0.05 μg/well of recombinant anthrax PA in PBS or 0.1 μg/well of recombinant anthrax PA in carbonate buffer. After removing the coating antigen, wells were blocked with 100-150 μl of blocking solution (2% skim milk in PBS containing 0.05% Tween 20) for 60-90 min at room temperature to prevent nonspecific binding. After three washes with PBS containing 0.05% Tween 20, undiluted BALF samples (two- to three-fold) or serum samples pre-diluted 1:500 in blocking buffer were added and incubated for 90 min at either room temperature or 37°C. Following sample incubation, plates were washed three times, then incubated with horseradish peroxidase (HRP)-conjugated secondary antibodies (goat anti-mouse IgG; GeneTex, 1:5000 dilution or IgA; SouthernBiotech, 1:2000 dilution) for 90 min at either room temperature or 37°C. Plates were washed again, and antibody binding was visualized by adding the chromogenic substrate 3,3’,5,5'-tetramethylbenzidine (TMB; BD, 555214). The reaction was stopped by adding an equal volume of 2N sulfuric acid, and absorbance was measured at 450 nm using a microplate reader.

### Flow Cytometry

Flow cytometry was conducted to evaluate germinal center (GC) B cell responses in mediastinal lymph nodes following immunization. Mediastinal lymph nodes (medLNs) harvested from immunized mice were mechanically dissociated to generate single-cell suspensions. The resulting cell suspensions were incubated with Fc-block (anti-CD16/32) to prevent non-specific antibody binding and subsequently stained with a panel of fluorochrome-conjugated or biotinylated antibodies specific for the following markers: CD3 (clone 145-2C11), Fas (Jo2), IgG1 (A85-1), IgG2a/2b (R2-40), and CD138 (281-2) from BD Biosciences, and CD19 (clone 6D5) , GL7 (GL7), IgD (11-26c.2a) from BioLegend. Streptavidin-conjugated fluorophores were used for biotinylated antibodies.

Cells were stained for 30 min at 4°C in staining buffer (PBS supplemented with 2% fetal bovine serum and 0.1%sodium azide), followed by washing steps. After staining, the cells were analyzed using flow cytometer machines (LSR Fortessa, BD Biosciences; Cytek Northern Lights, Cytek Biosciences, USA). Data acquisition and analysis were conducted using FlowJo software version 10.10.0 (FlowJo LLC). GC B cells were identified as CD19+ Fas+ GL7+ cell populations. Appropriate single-stain controls and fluorescence minus one (FMO) controls were employed to ensure accurate gating and data interpretation. Results were analyzed using FlowJo software (v.10.10.0).

### Toxin Neutralization Assay (TNA)

The neutralizing activity of PA-specific antibodies was evaluated using a toxin neutralization assay (TNA) with the murine macrophage cell line J774A.1 (ATCC TIB-67). Cells were cultured in Dulbecco's Modified Eagle Medium (DMEM; Cytiva) supplemented with 10% heat-inactivated fetal bovine serum, 1% penicillin-streptomycin, L-glutamine, and sodium pyruvate. Cells were harvested and seeded at a density of 4 × 10^4^ cells/well into 96-well flat-bottom plates and incubated overnight at 37°C in a 5% CO2 incubator.

Serial dilutions of serum or bronchoalveolar lavage fluid (BALF) samples were prepared separately in a 96-well plate. These diluted samples were incubated for 30 min at 37°C with anthrax lethal toxin (LeTx; composed of 50 ng/ml protective antigen [PA] and 50 ng/ml lethal factor [LF]). Following this incubation, the cell culture medium was removed, and the pre-incubated toxin-antibody mixtures were added directly to the macrophage cells. After a 4-hour incubation period at 37°C, cell viability was assessed using the Quanti-MAX WST-8 Cell Viability Assay Kit reagent (Biomax, QM2500), added to each well and incubated for an additional hour at 37°C. Absorbance was measured at 450 nm using a microplate reader.

### Statistical Analysis

Statistical analyses were conducted using GraphPad Prism software (GraphPad Software, USA). Differences among experimental groups were assessed using a one-way or two-way analysis of variance (ANOVA), depending on the experimental design, followed by Tukey’s multiple comparisons test to identify significant differences between specific groups. Comparisons between two samples were conducted using an unpaired two-tailed t-test. The Mann-Whitney U test was employed to compare AUC and median inhibitory concentration (IC50) values in toxin neutralization assays between groups. All data are presented as mean ± standard error of the mean (SEM), with statistical significance defined as *p* < 0.05.

## Results

### Optimization of the Promoter and Anchor System for Antigen Display on the Surface of *L. plantarum*

Efficient display of target antigens on bacterial surfaces requires an appropriate anchor system that effectively immobilizes proteins to the bacterial cell wall. In Gram-positive bacteria, the N-terminal transmembrane (NTT), LysM, and LPxTG anchor systems are the most extensively characterized and widely utilized methods for protein surface display [21, 22]. To identify the optimal anchor system for antigen presentation using our *L. plantarum* strain, we systematically evaluated these three anchor systems (Fig. 1A). For this comparative analysis, we expressed the receptor-binding domain (RBD) of SARS-CoV-2, tagged with hemagglutinin (HA) as a model antigen, driven by the elongation factor Tu promoter. Immunostaining analysis clearly demonstrated that the LPxTG anchor provided superior antigen stability and more robust surface expression compared to the NTT and LysM anchor systems (Fig. 1A). Consequently, we selected the LPxTG anchor system for subsequent experiments.

Next, we optimized promoter efficiency to further enhance antigen expression levels. We compared the elongation factor Tu promoter to our laboratory-optimized GAPDH promoter by analyzing their antigen expression efficiency using immunofluorescence. The GAPDH promoter notably enhanced antigen expression compared to the Tu promoter (Fig. 1B). Based on these results, the combination of the optimized GAPDH promoter and the LPxTG anchor system was chosen to construct our final recombinant *L. plantarum* vector (Fig. 1C). This optimized platform is expected to significantly improve antigen expression efficiency, thereby enhancing its potential as a nasal vaccine delivery system.

### Generation of Recombinant *L. plantarum* Expressing Anthrax-Protective Antigen (PA) Protein

To establish a recombinant *L. plantarum* strain capable of displaying the anthrax protective antigen (PA) on its surface, the full-length PA gene was first codon-optimized according to the codon usage patterns of *L. plantarum*. Additionally, a dendritic cell-targeting peptide (DCpep) [23] sequence was fused to the C-terminal region of the PA antigen (Fig. 1C). The resulting construct (PA-DCpep) was further modified by adding a C-terminal HA epitope tag and linked to the LPxTG anchor system, generating the final recombinant construct, termed PA-DCpep-HA_LPxTG (Fig. 1C). This construct was placed under the control of our laboratory-optimized GAPDH promoter in the recombinant plasmid PA_G1C256SB, which was subsequently transformed into *L. plantarum* (Fig. 1C).

### Verification of PA Antigen Display on the Surface of Recombinant *L. plantarum*.

**Fig. 2:**
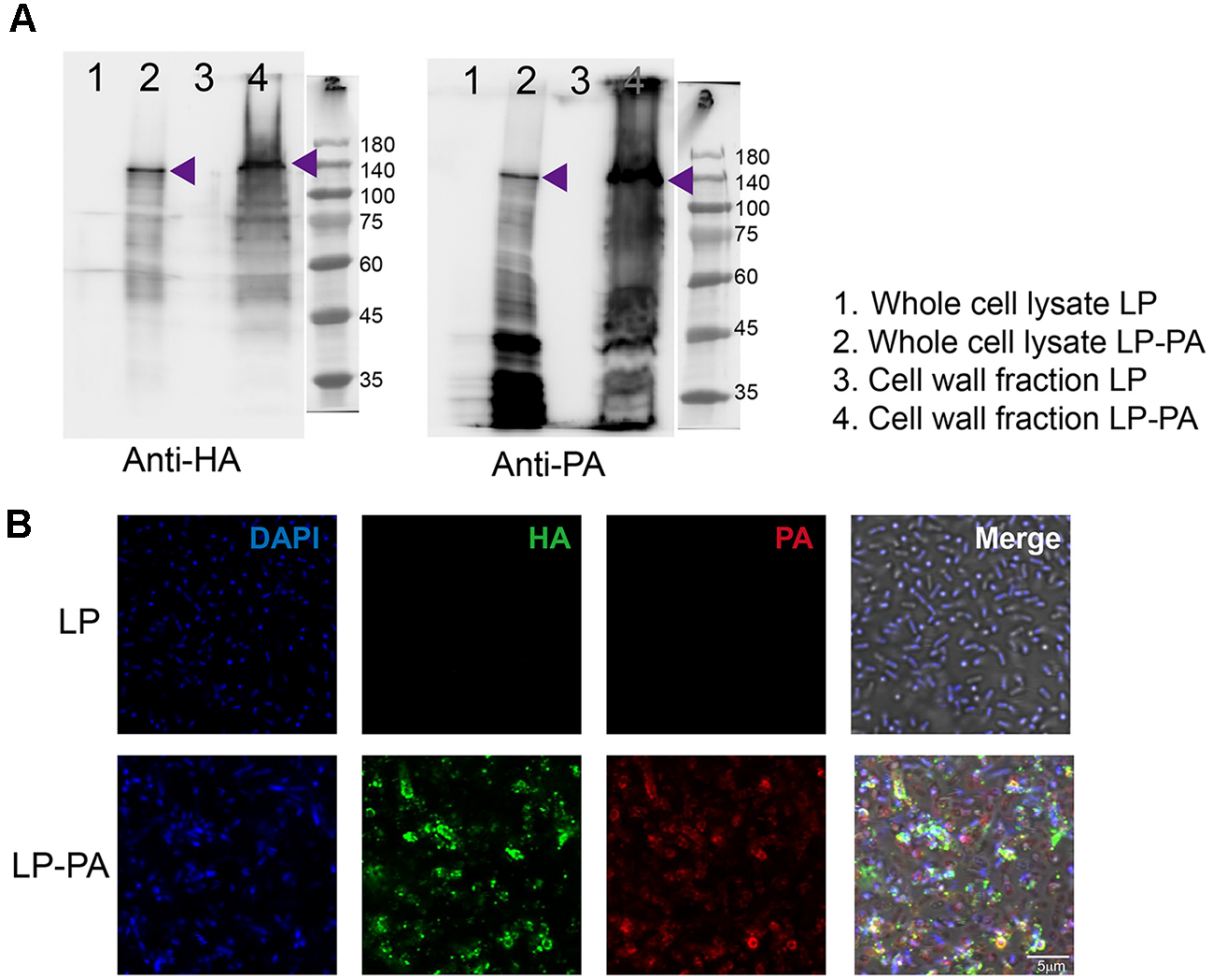
Verification of recombinant PA antigen expression and surface display in *L. plantarum*. (**A**) Western blot analysis confirming recombinant PA protein expression. The recombinant protective antigen (PA) protein was detected at the expected molecular weight in both whole-cell lysates and cell wall fractions of recombinant *L. plantarum* expressing PA (LPPA). No PA-specific signals were detected in the wild-type (WT) *L. plantarum* strain (LP), confirming antigen specificity. Detection was performed using anti-PA and anti-HA antibodies to verify the presence and integrity of the HA-tagged PA fusion protein. (**B**) Immunofluorescence staining of recombinant PA on *L. plantarum* cell surfaces. Anti-HA antibody staining was conducted under non-permeabilizing conditions to specifically detect extracellular surface-displayed proteins. Recombinant LP- PA exhibited distinct surface localization of HA-tagged PA antigen (green), while no signals were detected in WT controls (LP). Co-localization of signals from anti-PA (red) and anti-HA (green) antibodies confirmed efficient surface display of the recombinant PA antigen. Bacterial nuclei were counterstained with DAPI (blue). Scale bar: 5 μm.

To confirm successful expression of the recombinant PA antigen, we initially performed Western blot analysis. Results clearly demonstrated that the recombinant PA protein was detected at the expected molecular weight in both the whole-cell lysate and the cell wall fraction of recombinant *L. plantarum*. Importantly, no PA expression was observed in the wild-type strain, indicating specificity and successful recombinant protein production (Fig. 2A). Detection using both anti-PA and anti-HA antibodies yielded identical band patterns, conclusively verifying the identity of the recombinant PA-DCpep-HA fusion antigen. We believe that the smeared bands below the expected size of PA are most likely degraded forms of PA, which are commonly observed in recombinant proteins expressed in *E. coli*. The potential impact of degraded PA proteins on immunogenicity was not examined in the scope of this study.

To further confirm the localization of PA antigen on the bacterial cell surface, immunofluorescence staining was performed. An anti-HA antibody was used under non-permeabilizing conditions to specifically detect extracellularly displayed antigens. Recombinant *L. plantarum* showed distinct cell-surface staining signals, while wild-type *L. plantarum* remained negative, confirming successful external antigen display (Fig. 2B). Moreover, staining with an anti-PA antibody showed precise co-localization with anti-HA signals on the cell wall, further validating that the PA antigen was efficiently and specifically anchored to the surface of recombinant *L. plantarum* (Fig. 2B). When both antibodies were applied simultaneously, the intensity of the green and red signals varied significantly among individual bacterial cells. For instance, some cells showed both signals clearly, while others displayed a stronger green or red signal. We believe this variability is primarily due to steric interference, where binding of the first antibody to PA may hinder the binding of the second antibody.

Collectively, these findings demonstrate that our recombinant expression system using *L. plantarum* effectively facilitates robust surface display of antigenic proteins, highlighting its potential as a mucosal vaccine platform.

### Nasal Boosting with Recombinant LP-PA Specifically Enhances Mucosal IgA Production

**Fig. 3:**
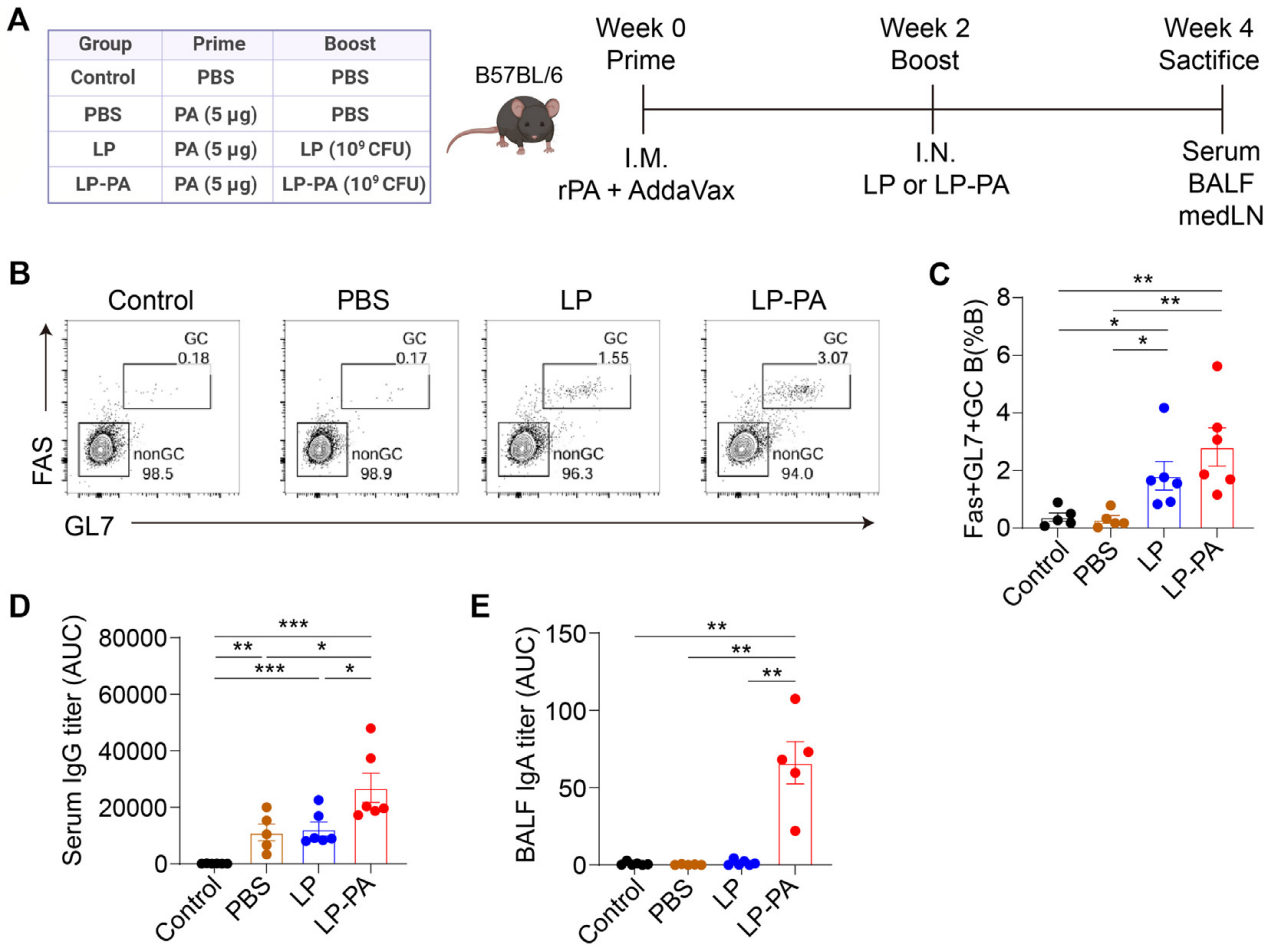
Nasal boosting with recombinant *L. plantarum* expressing PA (LP-PA) enhances germinal center responses and mucosal antibody production against anthrax protective antigen (PA). (**A**) Schematic overview of the immunization protocol. Mice were primed via intramuscular (I.M.) injection with purified recombinant PA protein and subsequently boosted intranasally (I.N.) with either recombinant *L. plantarum* expressing PA (LP-PA) or control *L. plantarum* (LP). Serum, bronchoalveolar lavage fluid (BALF), and mediastinal lymph nodes (medLN) were collected for immune analyses at week 4 post-priming. (**B, C**) Analysis of germinal center B cell responses in mediastinal lymph nodes by flow cytometry. (**B**) Representative flow cytometry plots illustrate the frequency of GC B cells identified as Fas+GL7+ within CD19+ B cells. (**C**) Quantitative analysis demonstrates a significant increase in GC B cell frequencies following nasal boosting with both LP and LP-PA compared to the control group, indicative of enhanced germinal center activation. (**D, E**) Antibody responses measured by ELISA. (**D**) PA-specific IgG antibody levels in serum. (**E**) Mucosal PA-specific IgA antibody levels in BALF. Data represent the mean ± SEM from two independent experiments (*n* = 5-6 mice per group). Statistical analyses were conducted using unpaired two-tailed t-test in (**C–E**). *p < 0.05, **p < 0.01, ***p < 0.001, ****p < 0.0001.

To evaluate whether nasal immunization with recombinant *L. plantarum* expressing anthrax protective antigen (LP-PA) can effectively induce protective mucosal antibody responses, we employed a prime-boost vaccination strategy validated in previous mucosal immunization studies [12, 13, 24]. Mice were initially primed intramuscularly (IM) with recombinant PA protein and subsequently boosted intranasally (IN) with 1 × 10^9^ colony-forming units (CFU) of either LP-PA or non-antigen-expressing control *L. plantarum* (LP) for two consecutive days. A phosphate-buffered saline (PBS) control group was included for comparison (Fig. 3A).

We first analyzed germinal center (GC) B cell responses in mediastinal lymph nodes using flow cytometry. Nasal boosting with LP-PA significantly enhanced the frequency of GC B cells (CD19+Fas+GL7+) compared to PBS control mice (Fig. 3B, C). Notably, nasal administration of non-antigen-expressing *L. plantarum* (LP) also moderately increased GC B cells, suggesting a potential adjuvant effect of the bacterial vector alone (Fig. 3B and 3C).

Next, we assessed antigen-specific systemic and mucosal antibody responses using ELISA. Serum PA-specific IgG titters increased significantly after IM priming in all primed groups, with LP-PA nasal boosting further augmenting these systemic antibody levels (Fig. 3D). These findings suggest that nasal boosting with antigen-displaying LP-PA can further augment systemic antibody responses initially induced by IM priming. Critically, mucosal IgA responses were exclusively induced in bronchoalveolar lavage fluid (BALF) from mice receiving LP-PA nasal boosting, while mice receiving PBS or non-antigen-expressing LP failed to develop detectable mucosal PA-specific IgA (Fig. 3E).

### Mucosal IgA Induced by LP-PA Nasal Boosting Confers Protection against Anthrax Toxin

**Fig. 4:**
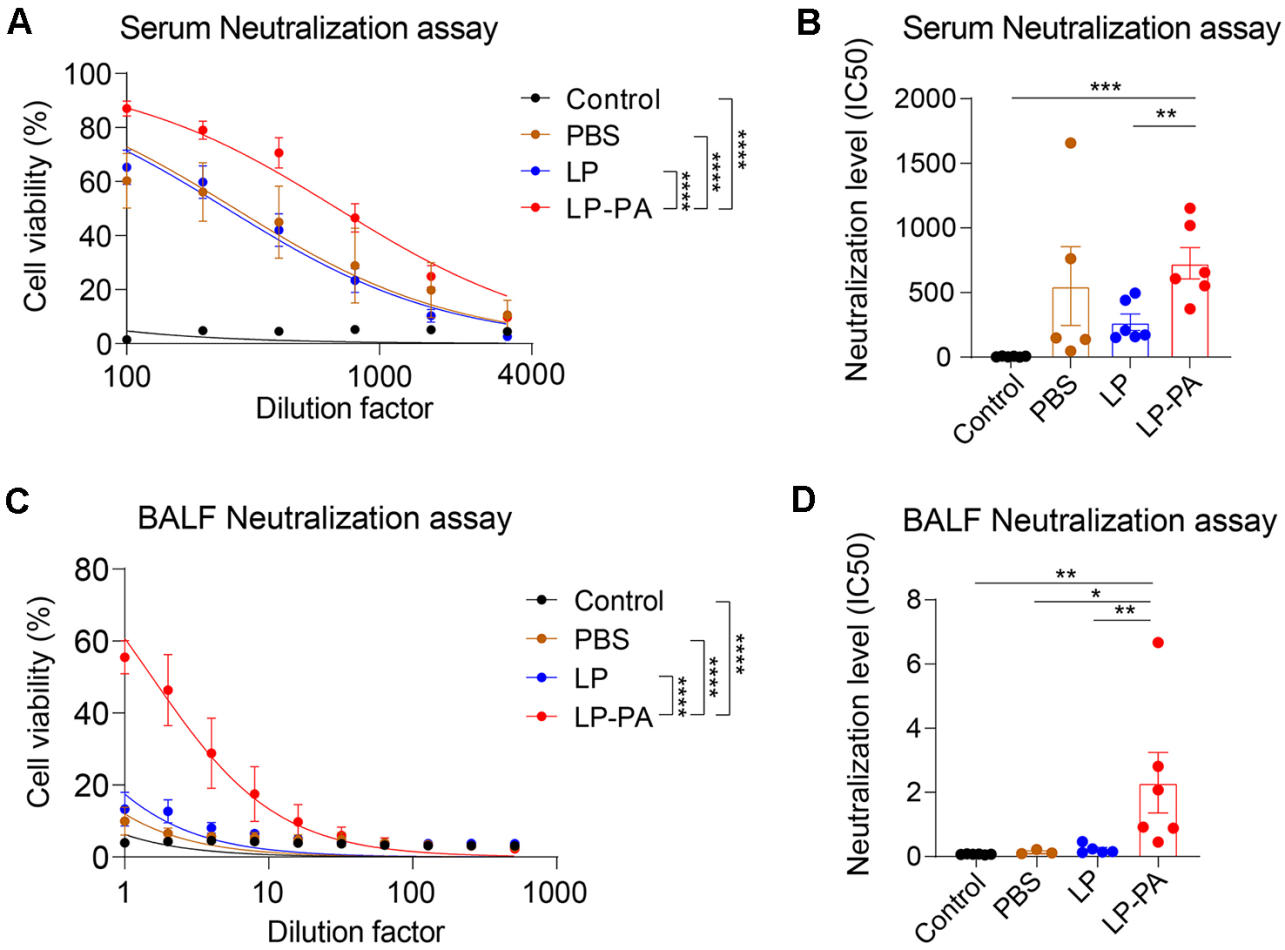
Functional evaluation of PA-specific antibody neutralizing activity by toxin neutralization assay (TNA). (**A, B**) Toxin neutralization activity of serum antibodies. (**A**) Cell viability (%) of J774A.1 macrophages treated with anthrax lethal toxin (LeTx) pre-incubated with serially diluted serum samples from immunized mice. (**B**) Comparison of median inhibitory concentration (IC50) values of serum from each experimental group. (**C, D**) Toxin neutralization activity of mucosal antibodies in bronchoalveolar lavage fluid (BALF). (**C**) Cell viability (%) of macrophages treated with LeTx preincubated with serially diluted BALF samples. (**D**) Comparison of IC50 values of BALF from each group. BALF from mice boosted nasally with recombinant *L. plantarum* expressing PA (LP-PA) showed significant and dose-dependent neutralizing activity, whereas BALF from control mice boosted with *L. plantarum* alone (LP) exhibited no protective effect. Data represent mean ± SEM from two independent experiments (*n* = 3-6 mice/group). Statistical analysis was performed using two-way ANOVA with Tukey’s post hoc test (**A, C**) or Mann-Whitney U test (B, D). *p < 0.05, **p < 0.01, ***p < 0.001, ****p < 0.0001.

To evaluate the functional relevance of these antibodies, toxin neutralization assays (TNA) were conducted using the J774A.1 murine macrophage cell line. Serum from all primed groups neutralized anthrax lethal toxin (LeTx) comparably, confirming robust systemic protection conferred by IM priming (Fig. 4A and 4B). Importantly, BALF from the LP-PA boosted group uniquely exhibited significant neutralizing activity against LeTx-mediated cytotoxicity in a dose-dependent manner, whereas BALF from all other groups lacked protective function (Fig. 4C and 4D). These results emphasize the specificity and functional importance of mucosal IgA antibodies elicited by LP-PA nasal boosting at the mucosal site of pathogen entry.

Collectively, our findings demonstrate that nasal administration of recombinant LP-PA following systemic priming potently induces protective mucosal IgA antibodies, specifically neutralizing anthrax toxin at respiratory mucosal surfaces. Thus, LP-PA nasal boosting represents a strategically advantageous approach for inducing route-specific protective immunity, underscoring its significant potential in developing mucosal vaccines against inhalational anthrax.

## Discussion

Anthrax, caused by the soil bacterium *Bacillus anthracis*, is a severe infectious disease affecting both animals and humans [1]. Human infection occurs primarily through contact with spores or consumption of contaminated food. Among the various forms-cutaneous, gastrointestinal, and inhalational anthrax—the inhalational form is notably the most severe, with rapid progression and high mortality rates if not treated promptly [4]. Due to its potent lethality and ease of aerosol dissemination, anthrax has been historically recognized as a potential bioweapon, with concerns regarding possible stockpiling by countries including North Korea [25]. Despite extensive research and development efforts, there remains a critical need for an effective, practical anthrax vaccine.

The current anthrax vaccines, primarily based on the protective antigen (PA), offer protection in animal models and have been widely studied for human vaccination [26, 27]. However, these vaccines require multiple intramuscular injections over prolonged periods, limiting their practicality for rapid and widespread deployment. For instance, the FDA-approved Anthrax Vaccine Adsorbed (AVA) necessitates six doses over 18 months and annual boosters thereafter [6, 7]. Additionally, although these vaccines are effective against systemic infection, their ability to induce mucosal immunity—critical for preventing inhalational anthrax—is minimal. Thus, developing a vaccine strategy that induces robust mucosal immunity is a high priority for enhancing protection against airborne pathogens.

In this study, we addressed these limitations by developing a *L. plantarum* platform expressing recombinant PA on its cell surface for nasal vaccine delivery. Our data demonstrate successful antigen expression on the bacterial surface and effective induction of mucosal immunity, specifically evidenced by PA-specific IgA production in respiratory mucosa. Importantly, our toxin neutralization assay confirmed that antibodies elicited by nasal boosting with recombinant *L. plantarum* efficiently neutralize anthrax lethal toxin.

These findings suggest that nasal immunization using recombinant *L. plantarum* expressing PA represents a significant advancement over existing vaccination strategies by effectively stimulating mucosal immunity. Consequently, this method could serve as a valuable booster platform for existing systemic vaccines, substantially reducing the logistical complexity and enhancing user compliance by minimizing invasive procedures.

Future studies should include evaluating this nasal vaccine in a robust animal infection model using attenuated strains, such as the Sterne strain [28], to confirm protective efficacy against actual anthrax infection. Additionally, evaluating long-term immune memory is essential for developing an effective vaccine strategy. Therefore, further studies on the durability of the mucosal immune response induced by our nasal boosting method will be necessary.

Moreover, further development and refinement of this platform into an aerosolized or spray formulation could enhance its applicability, allowing easy and rapid administration even under emergency circumstances such as warfare or bioterrorism threats. Ultimately, our novel nasal booster vaccine has the potential to revolutionize current anthrax vaccination practices by providing enhanced protection and facilitating easier deployment for mass immunization.
